# Dynamic phase coexistence in glass–forming liquids

**DOI:** 10.1038/srep11770

**Published:** 2015-07-09

**Authors:** Raffaele Pastore, Antonio Coniglio, Massimo Pica Ciamarra

**Affiliations:** 1 CNR–SPIN, Dipartimento di Scienze Fisiche, Universitá di Napoli Federico II, Italy; 2 Division of Physics and Applied Physics, School of Physical and Mathematical Sciences, Nanyang Technological University, Singapore

## Abstract

One of the most controversial hypotheses for explaining the heterogeneous dynamics of glasses postulates the temporary coexistence of two phases characterized by a high and by a low diffusivity. In this scenario, two phases with different diffusivities coexist for a time of the order of the relaxation time and mix afterwards. Unfortunately, it is difficult to measure the single-particle diffusivities to test this hypothesis. Indeed, although the non-Gaussian shape of the van-Hove distribution suggests the transient existence of a diffusivity distribution, it is not possible to infer from this quantity whether two or more dynamical phases coexist. Here we provide the first direct observation of the dynamical coexistence of two phases with different diffusivities, by showing that in the deeply supercooled regime the distribution of the single-particle diffusivities acquires a transient bimodal shape. We relate this distribution to the heterogeneity of the dynamics and to the breakdown of the Stokes-Einstein relation, and we show that the coexistence of two dynamical phases occurs up to a timescale growing faster than the relaxation time on cooling, for some of the considered models. Our work offers a basis for rationalizing the dynamics of supercooled liquids and for relating their structural and dynamical properties.

Glass forming systems have a spatially and temporally heterogeneous dynamics^1^ as revealed, for instance, by the time evolution of the Van Hove (vH) distribution function. This is the probability distribution that a particle has moved of a distance *r* along a fixed direction at time *t*, and is a Gaussian with variance *Dt* if particles move with a constant diffusion coefficient *D*. Conversely, in glass formers the vH distribution has a temporary non-Gaussian shape^2^,^3^,^4^,^5^, that indicates the temporary coexistence of particles with different diffusion coefficients. It has been suggested^6^,^7^,^8^,^9^,^10^,^11^ that this dynamical heterogeneity reflects the transient coexistence of two phases with different dynamical features, commonly indicated as the ‘fast’ and as the ‘slow’ phase. However, in equilibrium systems it has not yet been identified a dynamical order parameter with a transient bimodal probability distribution, which would support the existence of two coexisting phases; indeed, up to now a dynamical order parameter with a bimodal distribution has only been identified in structural glasses driven out of equilibrium introducing a field pinning some of the particles^9^, and thus inducing the two phases, or more complex constraints on the relaxation dynamics^10^. Because of this, in equilibrium supercooled liquids the ‘fast’ and the ‘slow’ phase are usually empirically defined, for instance by considering as ‘fast’ 5% particles, chosen to be the ones with the largest displacement^12^. These empirical criteria are used because the vH distribution cannot have a bimodal or multimodal shape allowing for the clear identification of different coexisting phases. Indeed, if phases with different diffusivities coexist, then the vH distribution will be the weighted sum of different Gaussian functions, all centered in *r* = 0, and will thus have a single maximum. This clarifies that, in order to investigate whereas two or more dynamical phases coexist, one should investigate the diffusivity distribution, not the vH distribution. Unfortunately, it is difficult to measure the single particle diffusivities; in particular, only stationary diffusivity distributions can be obtained via a direct inversion of the vH distribution^13^,^14^.

Here we report the first measure of the time evolution of the single particle diffusion coefficient, for different model systems: the standard Kob–Andersen Lennard–Jones (KALJ) binary mixture^15^,^16^,^17^, a binary mixture of soft-spheres in two dimensions^18^, and the Kob–Andersen lattice gas model^19^,^20^. In the deeply supercooled regime, we find this distribution to temporarily acquire a bimodal shape, thus proving the transient coexistence of two distinct dynamical phases. In the long–time limit the two phases mix and the diffusivity distribution acquires the expected Gaussian shape, with a variance to mean ratio we show to be related to the breakdown of the Stokes–Einstein relation. For some of the considered models, this mixing time scale grows faster than the relaxation time on cooling.

We succeeded in measuring the single particle diffusion coefficient by exploiting the intermittent nature of the single particle motion in structural glasses^21^,^22^,^23^,^24^,^25^,^26^,^27^. Indeed, particles in a glass spend most of their time confined within the cages formed by their neighbors, seldom hopping to different cages. This allows to describe the dynamics through the continuous time random walk (CTRW) formalism, reviewed in the Appendix. In this framework, the diffusivity of each particle at a given time is proportional to its number of jumps, and the distribution of diffusivities is equivalent to the distribution of the number of jumps per particle. Accordingly, in the following we first show that the CTRW approach quantitatively describes the dynamics of the considered systems, when cages and jumps are identified using a recently developed paramete–free algorithm^18^. We discuss in detail the KALJ system to show that the CTRW approach quantitatively describes the relaxation dynamics of atomistic systems, not only of kinetic lattice models^28^,^29^. Then, we use this approach to measure the diffusivity distribution and to investigate its time evolution.

## Results

### CTRW description of the dynamics

**Figure 1:**
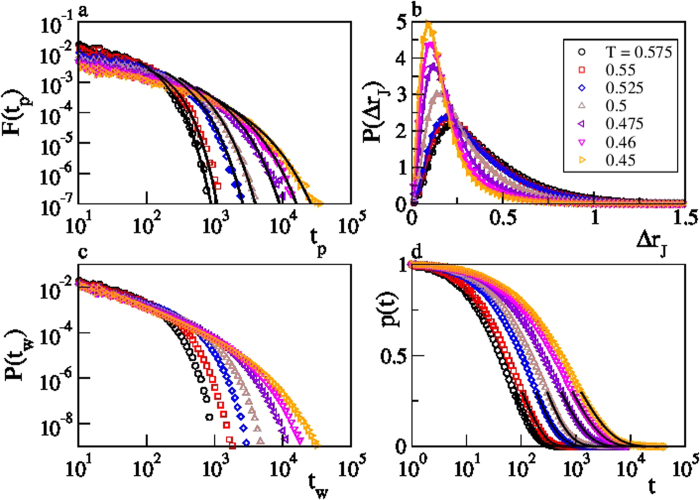
Persistence and cage–jump properties. Panels a,b and c show the probability distributions of the persistence time *t*_*p*_, of the jump length Δ*r*_*J*_, and of the waiting time *t*_*w*_. Panel d illustrates the decay of the persistence. Full lines in panel d are fits to stretched exponentials, while those in panel a are the corresponding predictions for *F*(*t*_*p*_) (see text). All data refer to species *a* of the KALJ mixture. Analogous results for species *b* are shown in Fig. S1.

In order to prove that the CTRW approach provides a quantitative description of the dynamics of the KA mixture, we have performed a careful analysis of the single particle cage–jump intermittent motion, for temperatures slightly above the mode–coupling one^15^,^16^,^17^, 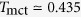. Figure 1a–c illustrate the distribution of the persistence time *F*(*t*_*p*_) and of the jump length *P*(Δ*r*), that fix the temporal and spatial features of the system in the CTRW approach, as well as the distribution of the time particles wait in their cages before making a jump, *P*(*t*_*w*_). No correlations between the persistence time and the jump length have been found, in agreement with the CTRW scenario. Panel d illustrates the decay of the persistence. At short times all jumps contribute to the decay of the persistence; we therefore observe 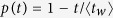, as 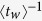 is the rate at which particles jump, and 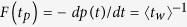. At long times 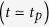 the persistence is found to decay with a stretched exponential, *p*(*t*) ∝ exp(−(*t*/*τ*)^*β*^). This implies *F*(*t*_*p*_) = −*dp*(*t*)/*dt* ∝ *τ*^−*β*^*t*^*β*−1^ exp(−(*t*/*τ*)^*β*^) as verified in Fig. 1a.

**Figure 2:**
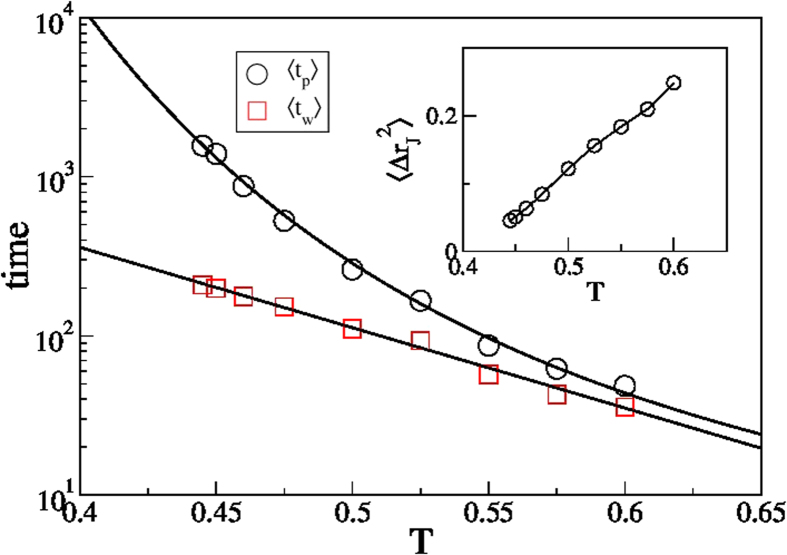
Cage–jump time and length scales. Temperature dependence of the average time particles persist in a cage before making the first jump, 〈*t_p_*〉, and of the average cage residence time, 〈*t_w_*〉. 〈*t_w_*〉 is well described by an Arrhenius 〈*t_w_*〉  ∝ exp(*A*/*T*) (full line). 〈*t_p_*〉 grows á super–Arrhenius law. The dashed line is a fit to 〈*t_p_*〉 ∝ exp(*A*/*T*^*B*^), with *B* = 2.4, but other functional forms, including the Vogel–Fulcher one, also describe the data. The inset illustrates the temperature dependence of the average jump length. The line is a guide to the eye. All data refer to species a of the KALJ mixture. Analogous results for species b are shown in Fig. S2.

**Figure 3:**
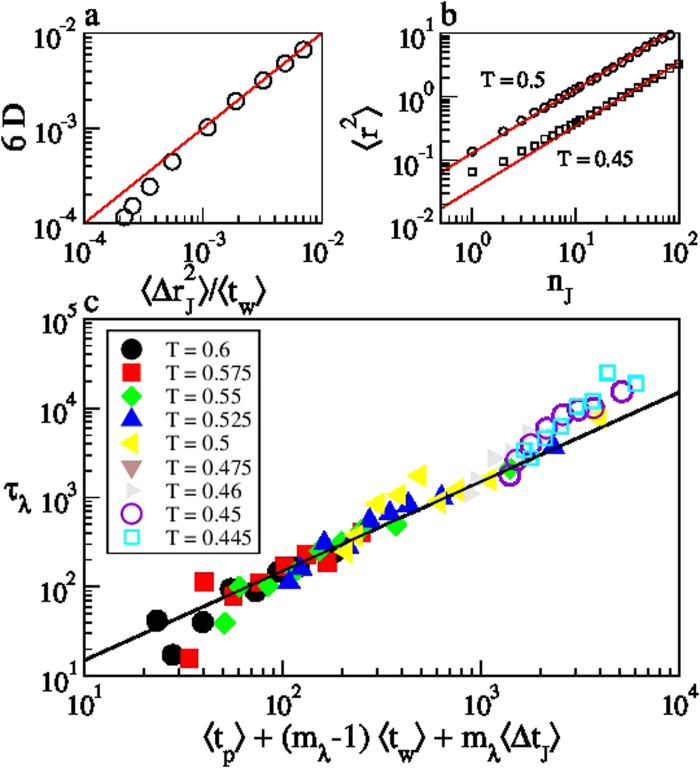
Structural relaxation and cage–jump properties. The diffusivity (panel a) and the relaxation time at a generic length scale *λ* (panel c) versus their predictions in the CTRW approach. Small deviations are observed at the lowest temperatures due to the emergence of a subdiffusive transient in the dependence of the mean square displacement on the number of jumps, as in panel b at *T* = 0.45. This indicates that successive jumps of a same particle becomes spatially correlated. All data refer to species *a* of the KALJ mixture. Analogous results for species *b* are shown in Fig. S3.

The temperature dependence of the main quantities characterizing the cage–jump motion is illustrated in Fig. 2. We observe the time scales 〈*t_w_*〉 and 〈*t_p_*〉 to have an Arrhenius and a super–Arrhenius behavior, respectively, and the average squared jump length to decrease on cooling. The temperature dependence of these quantities can be used to rationalize those of the diffusion coefficient *D* and of the structural relaxation time *τ*_*λ*_ at different wavelength *λ* (wavevector 2*π*/*λ*), which are commonly accessed experimentally. Indeed, in the CTRW approach it is easy to verify that 
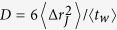
. Figure 3a illustrates that this relation is verified at the highest temperatures. Deviations emerge on cooling as subsequent jumps of a same particle becomes spatially correlated, as clarified by the subdiffusive dependence of the mean square displacement versus the number of jumps illustrated in panel *b*. The relaxation time *τ*_*λ*_ scales as the average time a particle needs to move a distance *λ*. Since in the CTRW approach subsequent jumps of a same particle are spatially uncorrelated, this time is that particles need to perform, on average, 
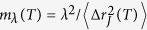
 jumps, and is fixed by the average time particles wait before making the first jump, 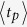, and the subsequent ones, 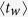, as well as by the average jump duration, 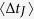:
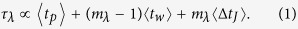
The last term is actually negligible at low temperatures, where 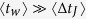
18. Figure 3c shows that this prediction agrees very well with the measured data in the investigated range of *λ*, with a coefficient of proportionality of the order of 1. As in the case of the diffusivities, small deviations are observed at the lowest temperatures. These results clearly demonstrate that 〈*t_w_*〉 and 〈*t_p_*〉 respectively correspond to the *β* and to the *α* relaxation time scales of structural glasses^28^,^30^,^31^, and confirm that the breakdown of the Stokes–Einstein (SE) relation, which is the increase of the product *τ*_*λ*_*D* on cooling, is mainly due to the increase of the 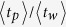 ratio, as in lattice model, but it is also affected by the temperature dependence of the jump length. Indeed, the length scale^28^ below which the breakdown of the SE relation occurs, 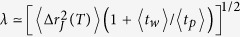, that is estimated equating the first two terms of the r.h.s. of Eq. 1, depends on the spatial features of the jumps.

### Diffusivity distribution

**Figure 4:**
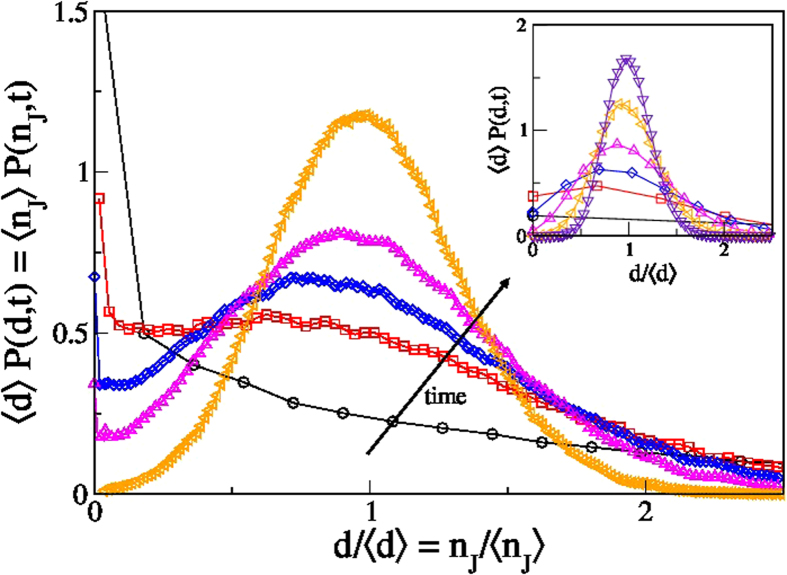
Diffusivity distribution. Probability distribution of the single particle diffusion coefficient at different time, rescaled by the average diffusivity, at *T* = 0.6 and *t* = 0.2, 1.4, 4, 5.2, 11, 25 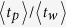 with 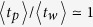 (inset), and at *T* = 0.45 and *t* = 0.65, 4.3, 7.7, 15, 29 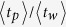 with 
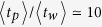
 (main panel). At low temperature and intermediate time, the distribution acquires a temporary bimodal shape with the maxima occurring at 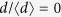 and 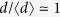, respectively. All data refer to species *a* of the KALJ mixture. Analogous results for species *b* are shown in Fig. S4.

The quantitative description of the relaxation dynamics through the statistical features of the cage–jump motion allows to exploit the CTRW approach to measure the distribution of the single particle diffusivities. Indeed, within the CTRW the diffusivity of particles that have performed *n*_*J*_ jumps at time *t* is 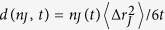. The diffusivity is therefore simply proportional to the number of jumps per unit time. Figure 4 illustrates the time evolution of the number of jumps per particle rescaled by the average number of jumps 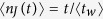, which coincides with the distribution of the single particle diffusion coefficient normalized by the average diffusion coefficient, 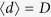. The inset and the main panel show results obtained at a high and at a low temperature, respectively. For 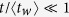, *P*(*d*; *t*) is peaked around zero as most particles have not jumped; conversely, in the infinite time limit the distributions have a Gaussian shape with average value 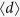. We observe that, at high temperature (Inset of Fig. 4), the distribution gradually broadens in time, and its maximum move from 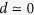 to 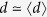. At low temperature (Main panel of Fig. 4), conversely, the distribution acquires a temporary bimodal shape before reaching the asymptotic Gaussian one. The bimodal shape proves the existence of a time window in which two phases of particles with different mobilities coexist. The two phases emerge because of the existence of two well separated timescales 〈*t_p_*〉 and 〈*t_w_*〉. Indeed the slow timescale, 〈*t_p_*〉, controls the value of the peak at *d* = 0, that equals the persistence correlation function, *P*(*d* = 0; *t*) = *p*(*t*). Conversely, the fast timescale, 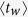, controls the average value of the distribution, as the position of the second maximum asymptotically occurs at 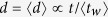. We stress that the phases with an high and with a low diffusion coefficient cannot be uniquely associated to particles that have moved over a small or over a large distance, respectively, as the average displacement of each particle is zero. This is why a bimodal distribution is not observed in the vH distribution function.

**Figure 5:**
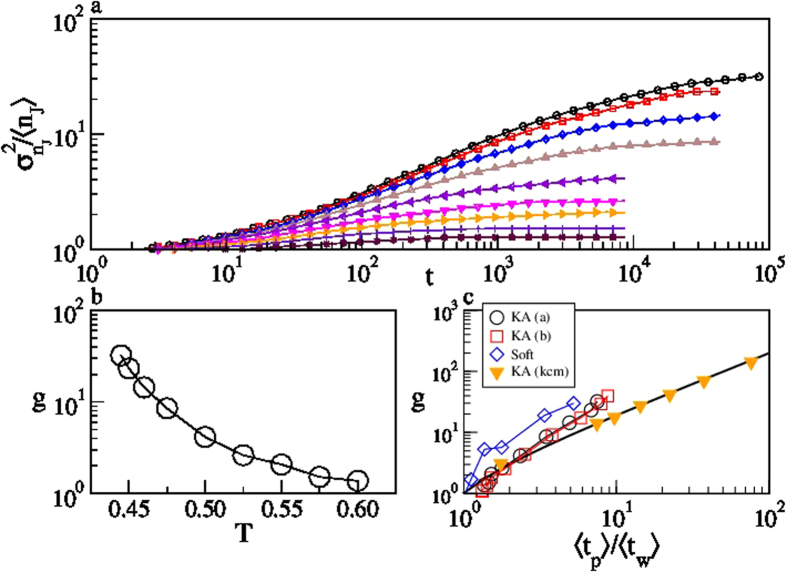
Variance to mean ratio of the distribution of the number of jumps per particle. Time evolution (**a**) of the variance to mean ratio of the distribution of the number of jumps per particle, and temperature dependence of its asymptotic value (**b**). Data refer to species *a*. Analogous results for species *b* are reported in Fig. S4. Panel c illustrates that, in the deeply supercooled regime, the asymptotic value scales as 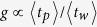, for both components of the KALJ mixture and for other model systems (see text). The full line is the CTRW prediction, 
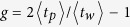
.

The time evolution of the distribution of the diffusivities gives further insights into the dynamics of the system. Indeed, Fig. 5a,b show that at long times the variance to mean ratio of *P*(*n*_*J*_; *t*) reaches a plateau value 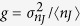, that grows on cooling. This plateau value can be related to the ratio of the two timescales 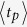 and 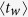. In fact, within the CTRW framework^32^,^33^ 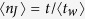 and 
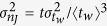
, where 
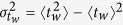
. Given the relation between the persistence time and the waiting time distributions^34^ (see Appendix), it follows 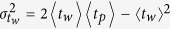 and thus 
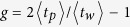
. We have verified this prediction considering, beside the KA model, also a binary mixture of harmonic spheres^18^ and the kinetically constrained Kob–Andersen three dimensional lattice gas model^19^,^20^, as illustrated in Fig. 5(inset). The lattice model confirms our predictions. The molecular dynamics simulations reproduce the asymptotically proportionality between *g* and 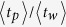, even though there are small deviations with respect to the CTRW prediction, suggesting the emergence of correlations between successive waiting times at low temperature.

A physical interpretation of the proportionality between *g* and 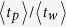 is obtained considering that a distribution with the long–time features of *P*(*n*_*J*_; *t*), i.e. a Gaussian distribution with variance 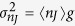, is obtained by randomly assigning the jumps to the particles, in group of *g* elements. Consistently, at high temperature *g* = 1 and *P*(*n*_*J*_; *t*) corresponds to that obtained by randomly assigning the jump to the particles, i.e. a Poisson distribution. The increase of *g* on cooling indicates that at low temperature one might observe, in the same time interval, some particles to perform *g* jumps, and other particles to perform no jumps at all, which clearly suggests 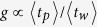.

### Spatial correlations

The CTRW approach does not make any assumption about the spatial correlations between the jumps of different particles. However, particularly in a facilitation scenario in which the jump of a particle facilitates the jumps of nearby particles, one expects these correlation to exist, and hence the two dynamical phases to be spatially segregated. Previous investigations of the spatio–temporal heterogeneities of structural glasses^1^ also suggest that this should be the case. Here we investigate these spatial correlation focusing on two correlation functions, both of them related to a scalar field associated to the number of jumps, 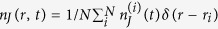. Note that *n*_*J*_(*r*, *t*)*tdr* is proportional to the average diffusion coefficient of the particles in the volume element *dr*.

First, we consider the spatial correlations between the particles that have not jumped at time *t*,
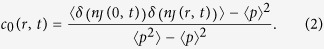
This equals the correlation function of the particles that have jumped, and thus of the particles that have moved of a distance greater than the jump length. *c*_0_(*r*,*t*) is therefore close to the commonly investigated four-point correlation function, at a wavelength related to the inverse jump length. Then we focus on the spatial correlations between the number of jumps, which is the spatial correlation of the diffusivity, considering the correlation function
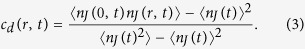


**Figure 6:**
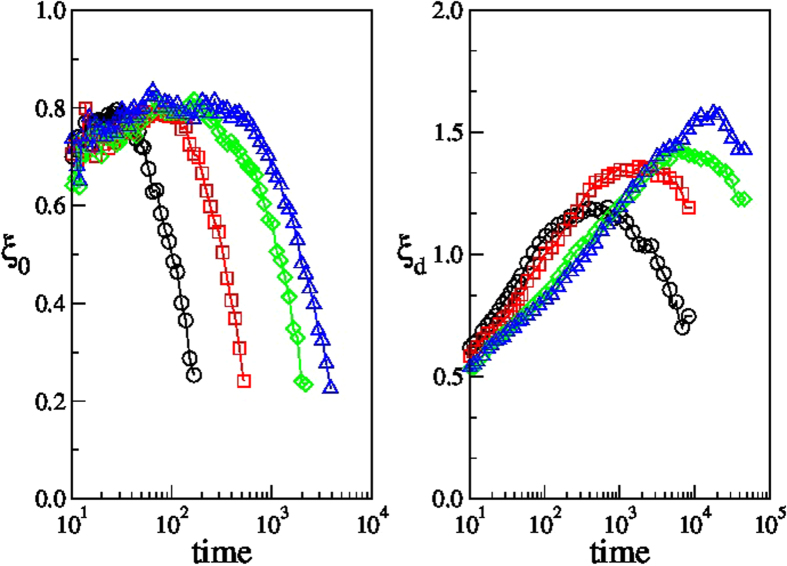
Spatial correlation lengths. Panel a illustrates the correlation length of the particles that have performed no jumps, panel b the diffusivity correlation length. From left to right: *T* = 0.55, 0.5, 0.46, 0.45.

**Figure 7:**
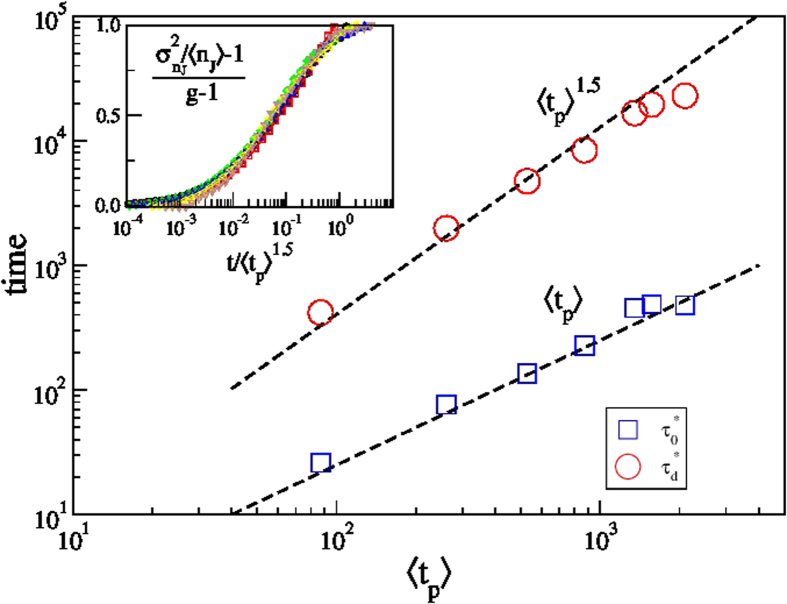
Temporal correlation lengths. Scaling of the times at which the correlation lengths *ξ*_0_ and *ξ*_*d*_ acquire their maximum value, with the persistence correlation time, 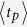. The inset shows the rescaling of the data of Fig. 5a, for temperatures *T* ≤ 0.55.

We find both correlations functions to decay exponentially, with correlation *ξ*_0_(*t*) and *ξ*_*d*_(*t*), respectively. Their time dependence is illustrated in Fig. 6, for selected temperatures. Both correlation lengths have a maximum as a function of time. We indicate with 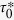 and 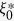, and with 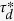 and 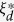, the time of occurrence and the value of the maxima of the two correlation lengths. As apparent from Fig. 6, both correlations length are small, as usual in structural glasses, and increases on cooling, 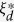 being much more temperature dependent than 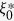. We characterize the temperature dependence of 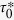 and 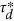 investigating their scaling with respect to the average persistence time, 〈*t*_*p*_〉. Figure 7 shows that 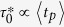, in agreement with previous results suggesting that the time of the maximum of the dynamical heterogeneities scale as the relaxation time. Conversely, we approximately find 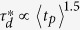. We note that the relation between 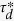 and 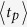 is model dependent, as for instance we observe 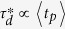 in the Kob–Andersen lattice gas model. Since 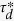 controls the diffusivity correlations, we expect it to also control the approach of the diffusivity distribution to its asymptotic Gaussian shape, and thus to be the time scale at which the variance to mean ratio 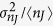 reaches its asymptotic value *g*, as in Fig. 5a. Indeed, the data of Fig. 5a are successfully rescaled when normalized and plotted versus 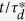, as in Fig. 7(inset).

The study of the time evolution of the diffusivity distribution and of the correlation between the single particle diffusivities allows to identify a new relaxation timescale, 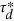. This grows faster than the persistence correlation time on cooling. The emerging physical scenario is as follows: the relaxation time of the system, as measured from the decay of scattering correlation functions, is essentially determined by 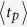, as in Eq. 1. However, on this time scale the diffusivities of the particles are spatially correlated, and the two dynamical phases are still coexisting, as the diffusivity distribution has not acquired its asymptotic normal shape. It is only on a time of the order of 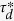 that all correlations are lost. On this time scale the diffusivity distribution has a Gaussian shape, and the diffusivities are spatially uncorrelated.

## Discussion

Our results show that the dynamics of supercooled liquids is characterized by the temporary coexistence of two phases with different diffusivities, one can reveal by describing the intermittent particle motion within the CTRW approach. The presence of these two phases is related to breakdown of the SE relation, that also fixes the variance to mean ratio of diffusivity distribution in the long time limit. The dynamical phase transition is characterized by a time scale 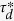, which is that after which the single particle diffusivities are both temporally and spatially uncorrelated. The temperature dependence of this time scale is model dependent, and we have observed it to scale as 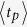
^1.5^ in the KALJ mixture. This result indicates that the mean squared displacement grows linearly in time for 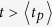, while the displacement distribution becomes Gaussian on a larger timescale, for 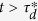. Accordingly, in between these two time scales the dynamics of the system is Fickian but not Gaussian^5^,^12^,^13^,^35^.

The clear identification of different dynamical phases might also allow to clarify the debated existence of correlation between the structural and the dynamical properties of supercooled liquids^11^,^36^,^37^,^38^,^39^,^40^. Indeed, these correlations have been looked for arbitrarily dividing the particles in a slow and in a fast phase, introducing a threshold on the particles’ displacements, and then considering how these phases are related to structural properties, such as Vöronoi volume, local order parameters, local elastic constants, or excess entropy. Our results suggest that the slow and the fast phase should correspond to phases with a high and a small diffusion coefficient, we have shown to be unambiguously identified.

## Methods

We have performed NVT molecular dynamics simulations^41^ of a *N* = 10^3^ standard Kob–Andersen 80:20 (*a*:*b*) binary Lennard–Jones (LJ) mixture^15^. Particles of species *i* and *j* interact via a LJ potential with energy scale *ε*_*ij*_ and length scale *σ*_*ij*_. Values are set as follow: *ε*_*aa*_ = 1.0; *σ*_*aa*_ = 1.0; *ε*_*ab*_ = 1.5; *σ*_*aa*_ = 0.8; *ε*_*bb*_ = 0.5; *σ*_*aa*_ = 0.88. Particles have the same mass *m*. *ε*_*aa*_, *σ*_*aa*_ and *m* are our units of energy, length and mass. For each temperature, we have first performed 200 simulations to obtain a smooth mean square displacement, from which we have extracted the Debye–Waller factor 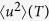 as in Ref. 42. We have then performed other 100 simulations to investigate the statistical features of the cage–jump motion as in Ref. 18: we associate to each particle, at each time *t*, the fluctuations *S*^2^(*t*) of its position computed over the interval [*t* − 10*t*_*b*_ : *t* + 10*t*_*b*_], with *t*_*b*_ ballistic time. The trajectory of each particle is segmented in cages and jumps, considering a particle to exit (enter) a cage as *S*^2^(*t*) becomes smaller (larger) than 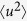. This procedure gives access to the duration of each cage, *t*_*w*_, and to duration Δ*t*_*j*_ and length Δ*r*_*J*_ of each jump. An analogous study has been performed for a 50 : 50 two dimensional mixture of particles interacting via a Harmonic potential^18^. In the case of the KA lattice kinetically constrained lattice glass model^19^, each particle movement is considered to be a jump.

### Appendix – CTRW

The Continuous Time Random Walk (CTRW) approach describes particle motion in supercooled liquids as a stationary isotropic walk process^43^. The temporal features of this process are fixed by the distribution *F*(*t*_*p*_) of the persistence time *t*_*p*_, which is the time particles wait before making their first step as measured from an arbitary *t* = 0 reference time. *F*(*t*_*p*_) is related to the distribution of the time *t*_*w*_ particles spend in their cages through the Feller relation^34^,^44^, 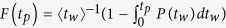. The spatial features are fixed by the distribution of the step size *P*(Δ*r*). The walk is assumed to be separable as no correlations between Δ*r* and *t*_*p*_ are considered. The relaxation dynamics is monitored by the persistence correlation function^20^,^28^,^29^,^31^,^45^ 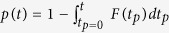, that equals the fraction of particles that has not moved up to time *t*. Accordingly the relaxation time *τ*, *p*(*τ*) = 1/*e*, scales as 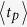; conversely, the diffusivity *D* scales as the number of steps per unit time, 
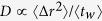
. While the CTRW approach assumes the waiting times of different particles to be uncorrelated, this assumption can be easily relaxed to capture the temporal heterogeneities of the dynamics. Indeed, if temporal correlations involve groups of *M* particles, then the fluctuation of the persistence of a *N* particle systems scales as 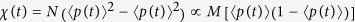, while its maximum value scales as 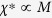.

## Additional Information

**How to cite this article**: Pastore, R. *et al*. Dynamic phase coexistence in glass-forming liquids. *Sci. Rep*. **5**, 11770; doi: 10.1038/srep11770 (2015).

## Supplementary Material

Supplementary Information
