# Supplementary material for: Dynamic phase coexistence in glass–forming liquids
Source: Sci Rep. 2015 Jul 9;5:11770. doi: 10.1038/srep11770 (PMC4496729; doi:10.1038/srep11770)
Supplement: Supplementary Information [file srep11770-s1.pdf]

Dynamic phase coexistence in glass–forming liquids

Raffaele Pastore, Antonio Coniglio, Massimo Pica Ciamarra

The main text reports results concerning the a species of the Kob–Anderson 80:20 (a:b) Lennard–Jones binary mixture. Here we show that species b behaves analogously. We thus illustrate in Figures S1–S5 the same quantities we have studied in Figures 1–5 of the main text, but this time present data concerning species b.

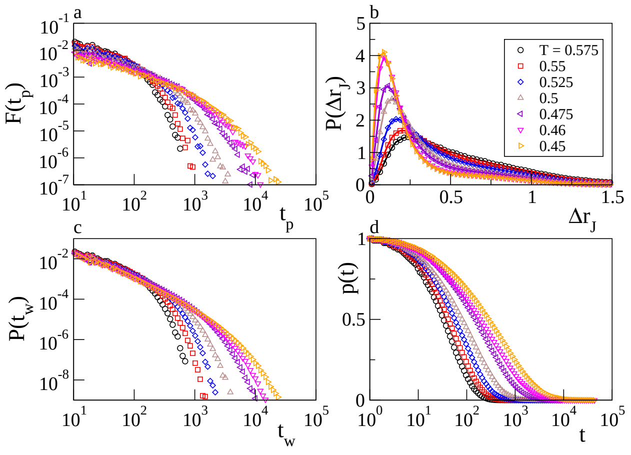

Figure S1: **Persistence and cage–jump properties.** Panels a,b and c show the probability distributions of the persistence time t_p, of the jump length Δr_J, and of the waiting time t_w. Panel d illustrates the decay of the persistence. All data refer to species b of the KA LJ mixture. Analogous results for species a are shown in Fig. 1 of the main text.

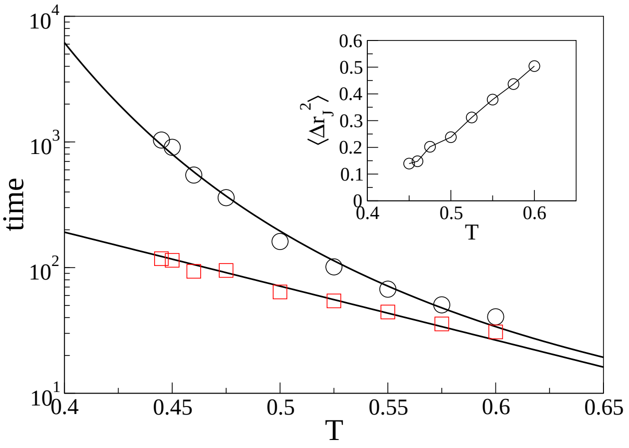

Figure S2: **Cage–jump time and length scales.** Temperature dependence of the average time particles persist in a cage before making the first jump, $\langle t_p \rangle$, and of the average cage residence time, $\langle t_w \rangle$. $\langle t_w \rangle$ is well described by an Arrhenius $\langle t_w \rangle \propto \exp(A/T)$ (full line). $\langle t_p \rangle$ grows á super–Arrhenius law. The dashed line is a fit to $\langle t_p \rangle \propto \exp\left(A/T^B\right)$, with $B = 2.2$, but other functional forms, including the Vogel–Fulcher one, also describe the data. The inset illustrates the temperature dependence of the average jump length. The line is a guide to the eye. All data refer to species b of the KA LJ mixture. Analogous results for species a are shown in Fig. 2 of the main text.

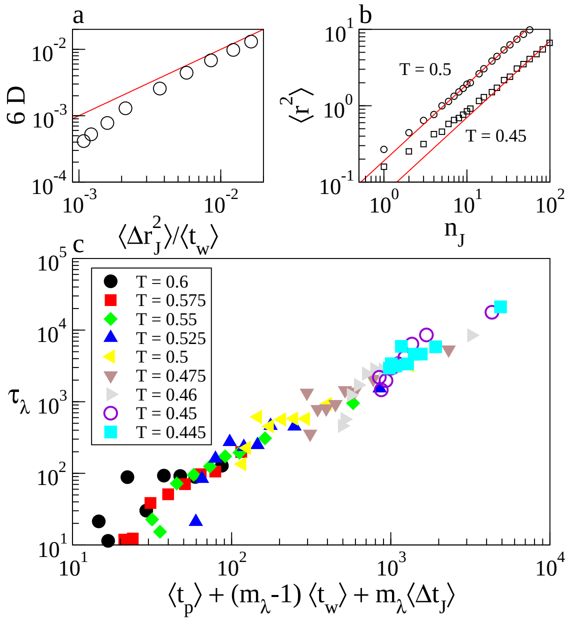

Figure S3: **Structural relaxation and cage–jump properties.** The diffusivity (panel a) and the relaxation time at a generic length scale λ (panel c) versus their predictions in the CTRW approach. Small deviations are observed at the lowest temperatures due to the emergence of a subdiffusive transient in the dependence of the mean square displacement on the number of jumps, as in panel b at $T = 0.45$. This indicates that successive jumps of a same particle becomes spatially correlated. All data refer to species b of the KA LJ mixture. Analogous results for species a are shown in Fig. 3 of the main text.

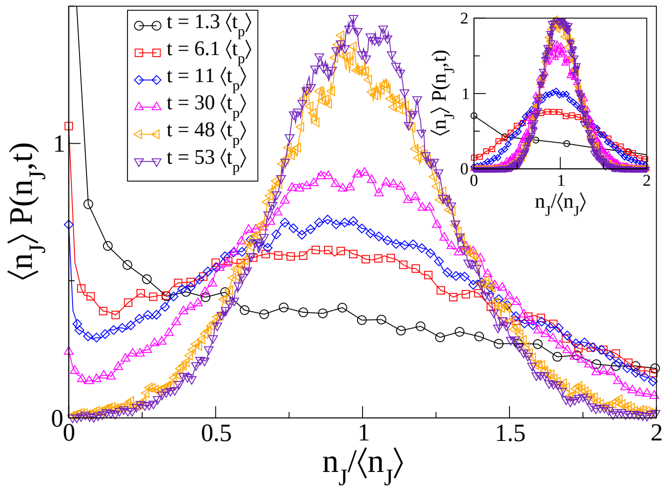

Figure S4: **Distribution of the number of jumps per particle.** Probability distribution of the number of jumps per particle at different different times, at $T = 0.5$ (inset) and at $T = 0.45$ (main panel). At low temperature, the distribution acquires a temporary bimodal shape. All data refer to species b of the KA LJ mixture. Analogous results for species a are shown in Fig. 4 of the main text

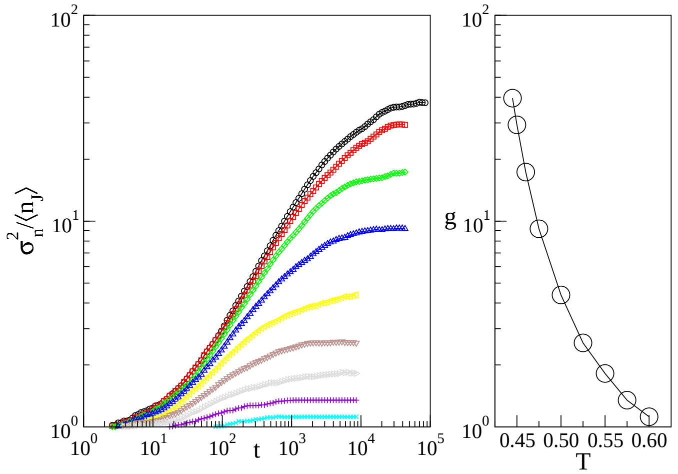

Figure S5: **Variance to mean ratio of the distribution of the number of jumps per particle.** Time evolution of the variance to mean ratio of the distribution of the number of jumps per particle (left panel), and temperature dependence of its asymptotic value (right panel). Data refer to species b. Analogous results for species a are reported in Fig. 5.
